# Effect of Whole-Body Vibration Exercise on Pain, Disability, Balance, Proprioception, Functional Performance and Quality of Life in People with Non-Specific Chronic Low Back Pain: A Systematic Review and Meta-Analysis

**DOI:** 10.3390/jcm13061639

**Published:** 2024-03-13

**Authors:** Tasneem Zafar, Saima Zaki, Md Farhan Alam, Saurabh Sharma, Reem Abdullah Babkair, Shibili Nuhmani, Sujata Pandita

**Affiliations:** 1 Centre for Physiotherapy and Rehabilitation Sciences, Jamia Millia Islamia, Maulana Mohammad Ali Jauhar Marg, New Delhi 110025, India; tasneem.zafar1999@gmail.com (T.Z.); saimazaki@jmi.ac.in (S.Z.); farhanphysio@gmail.com (M.F.A.); panditasujata@gmail.com (S.P.); 2 Department of Physical Therapy, College of Applied Medical Sciences, Imam Abdulrahman Bin Faisal University, Dammam 34212, Saudi Arabiasnuhmani@iau.edu.sa (S.N.)

**Keywords:** low back pain, rehabilitation, sensory–motor therapy, tonic vibration reflex, non-invasive pain management

## Abstract

**Background:** Non-specific chronic low back pain (NSCLBP) presents significant treatment challenges due to its multifactorial nature. Whole-body vibration exercise (WBVE) has emerged as a potential therapeutic modality, offering benefits across various domains, including pain reduction, improved balance, and enhanced quality of life (QoL). The aim of this present systematic review and meta-analysis is to evaluate the effects of WBVE on pain, disability, balance, proprioception, functional performance, and QoL in individuals with NSCLBP. **Methods:** We comprehensively searched PubMed, Web of Science, Scopus, and CENTRAL databases from October 2023 to January 2024, including RCTs with a PEDro score of ≥5 for high-quality evidence. Outcome measures included pain intensity, Oswestry Disability Index (ODI) score, Roland–Morris Disability Questionnaire (RMDQ) score, balance, proprioception, functional performance (through a progressive iso-inertial lifting evaluation), and QoL (SF-36) in NSCLBP patients. The risk of bias was assessed using ROB-2, and the certainty of evidence for each outcome indicator was analyzed using GRADE. A meta-analysis was conducted using standardized mean differences (SMD) and mean differences (MD) for continuous outcomes. **Results:** Ten randomized controlled trials fulfilled the inclusion criteria for the systematic review, and nine were suitable for the meta-analysis. The qualitative synthesis revealed WBVE is effective in improving pain, disability, balance, proprioception, and functional performance and QoL. Further, the results of the quantitative review demonstrated WBVE significantly reduced pain [visual analogue scale: SMD = −0.81, 95% CI (−1.11, −0.50), I^2^ = 0%, *p* < 0.01], disability [ODI: MD = −3.78, 95% CI (−5.27, −2.29), I^2^ = 24%, *p* < 0.01]; RMDQ: MD = −1.43, 95% CI (−2.04, −0.82), I^2^ = 51%, *p* < 0.01], balance [SMD = −0.28, 95% CI (−0.52, −0.05), I^2^ = 0%, *p* = 0.02], and proprioception [SMD = −4.20, 95% CI (−7.50, −0.89), I^2^ = 99%, *p* = 0.01]. **Conclusions:** This review and meta-analysis indicate that WBVE significantly improves pain, disability, balance and proprioception in individuals with non-specific chronic low back pain. These findings suggest potential benefits of incorporating WBVE into the management strategies for NSCLBP.

## 1. Introduction

Low back pain (LBP) is a prevalent musculoskeletal condition worldwide, impacting nearly everyone at some point in their lifetime [1]. While most cases of LBP resolve within 8 to 12 weeks, approximately 15% progress into chronic lower back pain (CLBP) [2]. Influenced by a multitude of factors such as lifestyle, social demographics, occupation, psychological aspects, age, and gender, LBP presents a significant challenge in clinical settings [3]. Particularly, non-specific chronic low back pain (NSCLBP), where specific causes remain elusive in 85% of cases even after imaging and biochemical analysis, presents a unique treatment challenge [4].

NSCLBP, often leading to immobilization and subsequent muscle atrophy, significantly impacts spinal stabilization capabilities [5]. Additionally, individuals with NSCLBP exhibit compromised proprioception compared to their healthy counterparts [6]. Given the crucial role of proprioception in joint stability, movement control, and injury prevention [7,8], its impairment in LBP patients can have profound implications, such as delayed muscle reflexes and increased vulnerability to spinal injuries [9,10]. This situation escalates the risk of recurrence and intensifies pain and disability, thus highlighting the importance of proprioceptive training in LBP management [11,12].

In recent decades, whole-body vibration exercise (WBVE) has emerged as a promising alternative therapy. WBVE aims to enhance muscle strength and activity by leveraging neurogenic potentiation [13]. This exercise modality, facilitated through vibrating platforms that generate mechanical oscillations, is thought to improve neural factors such as muscle recruitment, synchronization, and proprioceptor response [14]. The vibrations produced by the equipment spread throughout the body, inducing muscle stimulation via vibratory tonic reflex, initiated by the rapid changes in muscle length detected by different proprioceptive organs, ultimately enhancing the frequency of motor-evoked potentials [14]. This enhances muscle spindle activity, resulting in the activation of the trunk muscle stretch response, thus activating and strengthening the muscles in the lower back [14,15].

Beyond its direct neuromuscular benefits, WBVE has been increasingly recognized for its holistic impact on individuals with NSCLBP. Research indicates that WBVE significantly reduces pain levels [16], likely through the modulation of pain perception mechanisms and the reduction of inflammatory markers [17], offering a non-pharmacological option for pain management in NSCLBP patients. Additionally, WBVE contributes to a decrease in disability scores [18], suggesting an improvement in the daily functional capacity of individuals suffering from NSCLBP. The enhancement in balance and proprioception can be attributed to the stimulation of sensory receptors and the central nervous system’s adaptation to the vibratory stimuli, which are essential for maintaining postural control and reducing the risk of falls [19]. Furthermore, WBVE has been shown to improve functional performance by increasing the range of motion and muscle power, facilitating the execution of daily activities and potentially leading to a more active and independent lifestyle [16,18,20]. The positive impact on quality of life (QoL) is likely a cumulative effect of improvements in pain, disability, balance, and functional performance, contributing to overall well-being and satisfaction with life [21,22]. These multifaceted benefits underscore the potential of WBVE as a comprehensive therapeutic modality for individuals with NSCLBP, supporting its incorporation into treatment plans aiming to address the complex needs of this population.

However, the effectiveness of WBVE in managing NSCLBP remains uncertain, partly due to inconsistencies in research practices. Studies in the field vary significantly in terms of the frequencies, amplitudes, and durations used in WBVE, and there is a lack of standardized protocols across these studies [23,24,25,26]. While there are existing systematic reviews examining the impact of WBVE on aspects like pain, proprioception, and disability, these often include studies of local vibration and do not offer a comprehensive meta-analysis [26,27]. Moreover, recent systematic reviews and meta-analyses have focused solely on the effects of WBVE on pain and function, without a broader scope [25]. In this review, we delve into the assessment of QoL and functional abilities, recognizing that LBP profoundly affects various aspects of life, from basic self-care to complex social, work, and functional performance, ultimately impacting overall QoL [18,28]. We introduce the evaluation of functional performance and QoL as a novel aspect in this systematic review. Additionally, unlike previous systematic reviews, we have not considered local vibration therapies in inclusion.

Building on the identified challenges and gaps in the existing literature, this systematic review and meta-analysis aims to bridge these divides by comprehensively evaluating the effects of WBVE on pain, disability, balance, proprioception, functional performance, and QoL in individuals with NSCLBP. By incorporating a broader spectrum of outcomes and including studies with varied vibration protocols, this review seeks to provide a holistic understanding of WBVE’s role in managing NSCLBP and offer comprehensive, evidence-based treatment strategies for clinical practice.

## 2. Methods

### 2.1. Registration

Considering the guidelines for Preferred Reporting Items for Systemic Reviews and Meta-Analyses (PRISMA), we carried out this systemic review and meta-analysis [29]. Our PROSPERO registration number is CRD42023471941.

### 2.2. Eligibility Criteria

We utilized the PICO (population, intervention, comparator, outcome) framework to guide our study selection criteria, ensuring a focused and systematic approach to identifying relevant research [30]. The population targeted in this review comprised human subjects diagnosed with NSCLBP persisting for at least three months [31]. The diagnosis of NSCLBP was based on the absence of specific underlying causes, confirmed through patient history and physical examination, and by ruling out specific conditions via imaging or laboratory tests if necessary [32]. This diagnosis is typically applied when back pain persists for more than 3 months without an identifiable cause [32]. We included participants over the age of 10 years, across all genders and ethnicities. The inclusion of younger participants in our study is informed by evidence indicating that the prevalence of spinal pain in adolescents steadily increases with age and closely approximates adult levels by the age of 18 [32,33].The primary intervention of interest was WBVE. We included studies that either used WBVE alone or WBVE in combination with an intervention. For comparators, the review considered studies that used non-WBVE treatments or control groups. The outcomes focused on were measures of pain, disability, balance, proprioception, functional performance, and QoL.

Eligibility for inclusion required that studies investigate the effects of WBVE on individuals with NSCLBP. Only randomized controlled trials with a PEDro score of 5 or higher were considered, ensuring methodological rigor. The WBVE intervention had to be applied for a minimum duration of two weeks to be included in the review [27]. We excluded studies involving participants with CLBP attributable to identifiable pathologies through diagnostic imaging or laboratory tests. Exclusions also encompassed case reports, abstracts, meta-analyses, systematic reviews, literature reviews, and conference papers to focus on primary research with direct intervention outcomes. Furthermore, studies were omitted if they involved the application of local vibration as the sole treatment or if the duration of vibration therapy was less than two weeks, ensuring a focus on interventions with potential for significant therapeutic effects.

### 2.3. Search Strategy

To ensure a comprehensive and systematic identification of studies examining the effects of WBVE on NSCLBP, we (TZ, SZ and MFA) implemented an extensive search across several major scientific databases. Our search strategy, initiated in October 2023, covered electronic databases including PubMed, Web of Science, Scopus, and the Cochrane Central Register of Controlled Trials (CENTRAL). This comprehensive approach extended through January 2024, ensuring the inclusion of the most current studies available. Our approach involved a strategic combination of search terms and Boolean operators to capture a broad spectrum of relevant studies. Key search words included “Whole-body vibration AND chronic low back pain”, “Whole-body vibration AND chronic low back pain AND disability”, “Whole-body vibration exercise”, “Whole-body vibration AND chronic low back pain AND balance”, “Whole-body vibration AND chronic low back pain AND proprioception”, “Whole-body vibration AND chronic low back pain AND functional performance”, and “Whole-body vibration AND chronic low back pain AND quality of life”. This methodology was replicated across all selected databases to ensure consistency and comprehensiveness in our search. We confined our search to studies that were conducted on human subjects and published in the English language, aligning with our inclusion and exclusion criteria. To supplement our electronic database search and ensure no significant studies were overlooked, we also manually reviewed the reference lists of all identified articles. This dual approach of electronic and manual searches allowed for a thorough exploration of the available literature on the topic.

### 2.4. Study Selection Criteria

The search terms were assessed for each database, and all retrieved studies were imported into a reference manager software (EndNote™ Desktop version 21, Clarivate Analytics) for consolidating results and removing duplicate articles. Two independent reviewers (TZ and SZ) analyzed the abstracts and titles of the articles to determine their eligibility for inclusion. Any discrepancies in their assessments were resolved through discussion and consensus with a third reviewer (SS). In cases where abstracts and titles did not provide sufficient information regarding eligibility, the articles were examined in full text based on inclusion and exclusion criteria. Additionally, when data were missing or clarification was needed, the study authors were contacted.

### 2.5. Methodological Quality Assessment

The methodological quality of the included studies was assessed using the Physiotherapy Evidence Database (PEDro) scale, which is a 10-item scoring scale where the internal validity of randomized control trials is assessed [34]. Studies receiving a score of less than 5 out of 10 were recognized as low-quality studies [35]. Two reviewers (TZ and SP) scored each study independently and then met with a third reviewer (SS) for the conclusion. To further enhance the methodological quality assessment, the Cochrane Risk of Bias 2 (RoB 2.0) assessment methodology was also employed.

The RoB 2.0 tool involves a detailed evaluation of bias across several domains. Each domain is evaluated for overall bias, with specific questions scored as “yes”, “probably yes”, “probably no”, “no”, or “no information”. These individual domain scores are then aggregated into an overall assessment of bias, categorized as “low”, “some concern”, or “high” based on a predefined algorithm [36]. To ensure consistency, two researchers (TZ and SZ) independently assessed the RoB 2.0 tool and resolved any discrepancies through discussion with third reviewer (RAB), arriving at a final evaluation through consensus.

### 2.6. Certainty of Evidence

The methodology for evaluating the certainty of evidence in this systematic review involved creating Summary of Findings (SoF) tables, which were initially drafted by two independent authors (TZ and SZ), and any differences between their assessments were reconciled by consulting a third author (RAB). The level of certainty for each predetermined outcome measure was appraised using the Grading of Recommendations, Assessment, Development, and Evaluation (GRADE) system. This approach considers five critical domains that may affect the confidence in evidence and the strength of recommendations: risk of bias, inconsistency, indirectness, imprecision, and publication bias. These domains are assessed using the GRADEpro Guideline Development Tool (https://gdt.gradepro.org/app/ (accessed on 5 February 2024)), a piece of software designed by experts at McMaster University and Evidence Prime [37]. Based on the evaluation across these domains, the evidence is categorized into one of four levels of certainty: high, moderate, low, or very low. This structured methodology ensures a standardized, transparent, and systematic assessment of the quality of evidence, which is vital for informing clinical decision making and guideline development [38].

### 2.7. Statistical Analysis

Relevant data for the outcome measures were extracted from all study groups at both the beginning and the end of the intervention by the two authors (TZ and SN), covering key outcome measures. Initial data points were collected, including the number of participants (*n*), mean, standard deviation, *p*-value, and 95% confidence intervals (CI), when available. For further analysis, Review Manager (RevMan 5.4, provided by The Cochrane Collaboration) was utilized. Due to the consistent methodology across studies for certain outcomes, a fixed-effect model was applied to compute mean differences (MDs) for QoL (SF-36), and disability scores [Oswestry disability index (ODI) and Roland–Morris disability questionnaire (RMDQ)], along with their 95% CIs. Conversely, for outcome measures like progressive iso-inertial lifting evaluation (PILE), visual analog scale (VAS), numerical pain rating scale (NPRS), balance and proprioception, where methodological approaches varied, a random-effects model was employed. This is per recommendations to perform a meta-analysis [39]. The effect size of the outcome variables was interpreted by the MD or SMD values, with a value of 0.2 signifying a small effect size, 0.2–0.5 depicting a medium effect size, and >0.5 reflecting a high effect size according to Cohen’s criteria [40]. The heterogeneity among studies was assessed using the I^2^ statistic, where heterogeneity was interpreted as low (0–40%), moderate (30–60%), high (50–90%), and very high (75–100%), ensuring a comprehensive evaluation of variability among study findings [39]. A value of *p* < 0.05 was considered statistically significant. This methodological approach ensures a robust analysis of the treatment effects of WBV on the specified outcomes, facilitating a comprehensive understanding of its efficacy and applicability in clinical settings.

### 2.8. Dealing with Missing Data

In instances of missing data in included studies, attempts were made to contact the trials’ authors to acquire the necessary details. Studies that failed to report mean change and SD or lacked sufficient information for these calculations were excluded from the meta-analysis.

## 3. Results

### 3.1. Study Identifications

**Figure 1:**
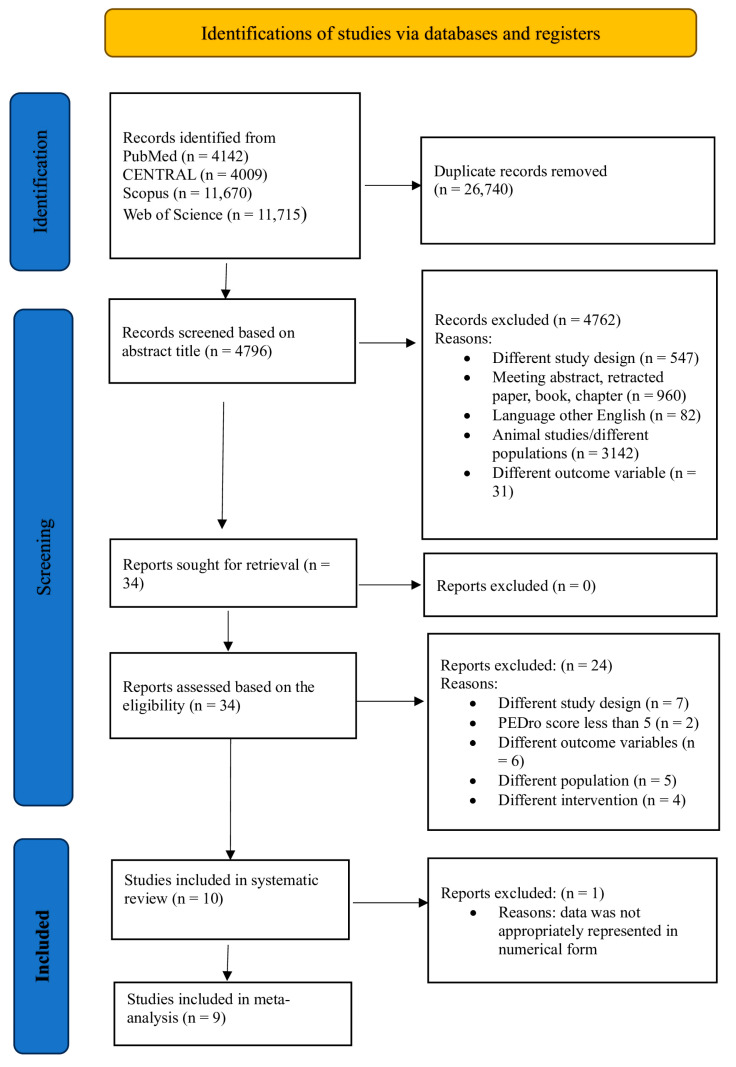
PRISMA flow chart.

In the systematic review and meta-analysis, the identification of studies began with a comprehensive search across multiple databases, leading to the initial identification of 31,536 articles (Table 1). Following the removal of 26,740 duplicate records, 4796 articles were screened based on titles and abstracts. Out of 4796 articles, 4762 articles were excluded due to various reasons such as incompatible study design (*n* = 547 articles); non-original reports like meeting abstracts, retracted papers, or book chapters (*n* = 960 articles); language barriers excluding non-English studies (*n* = 82 articles); the inclusion of animal studies or different target populations (*n* = 3142 articles); and research with divergent outcome variables (*n* = 31 articles). Consequently, this refinement process yielded 34 articles that were subjected to detailed full-text analysis based on eligibility criteria, leading to the further exclusion of 24 articles for various reasons such as different study designs (*n* = 7), PEDro scores less than 5 (*n* = 2), outcomes other than our inclusion criteria (*n* = 6), different populations (*n* = 5), and different interventions (*n* = 4). Ultimately, 10 articles met all the criteria for inclusion in our systematic review. However, one of these was omitted from the meta-analysis because it did not present quantifiable data adequately. As a result, nine studies were ultimately included in the meta-analysis (Figure 1).

### 3.2. Participants

A total of 10 studies were included, encompassing 828 participants at baseline. These studies collectively reported on individuals ranging in age from 10 to 70 years, all of whom were suffering from NSCLBP. The participant demographic across these studies was skewed towards a higher ratio of females compared to males. However, a precise gender distribution percentage could not be determined, as not all studies provided explicit data on the number of male and female participants.

### 3.3. Description of Interventions and Protocols

The interventions across the included studies showcased a wide range of WBVE protocols, targeting NSCLBP through various approaches [16,18,23,41,42,43,44,45,46,47]. Each study utilized specific vibration frequencies, amplitudes, durations, and session frequencies, tailored to explore the efficacy of WBVE in improving outcomes for NSCLBP patients. The detailed regimes included exercises like squats, lunges, bridges, and core stabilization, with intervention durations ranging from 2 to 12 weeks. These protocols were meticulously designed to assess the impact of WBVE on pain, disability, proprioception, balance, and QoL, highlighting the diversity in intervention strategies and the comprehensive nature of the treatment modalities employed (Table 2 and Table 3).

### 3.4. Outcome Measures

Pain: Pain intensity was measured using the VAS in four studies, although data from Cigdem Karacay et al. (2022) were excluded from the meta-analysis due to unsuitable VAS information [18]. For the study by Wang et al. (2019), which presented data in mean and confidence interval, we adjusted this to mean and standard deviation to maintain consistency in our analysis approach [23]. Furthermore, three studies evaluated pain using NPRS [41,43,44], among which two studies were included in meta-analysis [41,43].

Disability: Disability evaluation in our study involved the ODI and the RMDQ. Specifically, seven studies [16,23,42,43,45,46,47] utilized the ODI. Wang et al. (2019) provided their findings in means and confidence intervals, which were converted to mean and standard deviation for uniformity in analysis [23]. Additionally, Ruger et al. (2023) presented a comparative analysis of WBVE against two other treatments, necessitating an individualized approach for the meta-analysis [45]. Three studies [16,18,42] employed the RMDQ, with Cigdem Karacay et al. (2022) offering data at both post-treatment and three months post-treatment, enriching our longitudinal insight into disability outcomes.

Proprioception and balance: Proprioception was evaluated in two studies, with Jung et al. (2020) using a digital inclinometer for repositioning error analysis and Wang et al. (2019) applying a lumbar joint position test with an isokinetic dynamometer [23,41]. Wang et al. (2019) also differentiated proprioceptive measurements in flexion and extension movements, leading to duplicate data entries in the meta-analysis for a comprehensive assessment. Postural stability was assessed in five studies through various methods, including the Biodex balance system [16], MFT-S3-Check for evaluating stability index (SI) [46], an interactive balance system [45], and a fall index [47]. However, one study’s postural stability data were excluded from our meta-analysis due to its presentation not aligning with the required format for our analytical needs [42].

Functional performance: The evaluation of functional performance through the PILE test was specifically addressed in two studies within the review. One study meticulously recorded lifting capacity from ground to back and then from back to shoulder in the 8th and 12th weeks, ensuring a thorough analysis by incorporating all relevant data points separately [18]. In contrast, del Pozo-Cruz et al. (2011) provided a composite analysis of PILE performance before and after the intervention [16]. Isokinetic muscle strength was uniquely examined in one study by Cigdem Karacay et al. (2022), contributing a distinct perspective to the systematic review [18].

Quality of Life: Three studies QoL used the SF-36. However, comprehensive data were successfully retrieved from only two of these studies; Kaeding et al. (2017) and Wegner et al. (2019) provided insights into the SF-36′s mental and physical components, whereas Wang et al. (2019) detailed each of the eight SF-36 components individually, offering a broad spectrum of QoL outcomes [23,42,46].

A summary of all the eligible outcome measures included in the systematic review and meta-analysis is provided in the table (Table 4).

### 3.5. Risk of Bias across Outcomes

**Figure 2:**
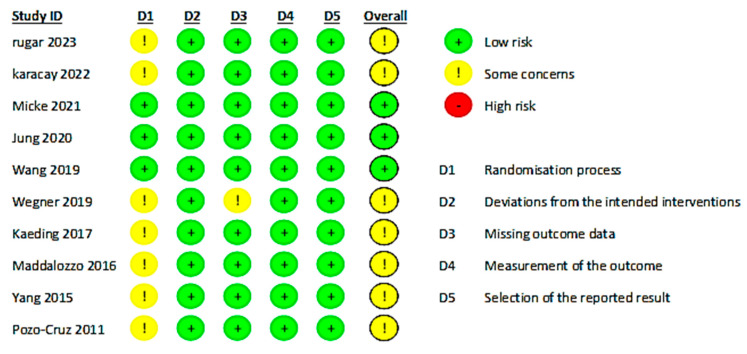
Risk of bias assessment for selected studies using Cochrane’s RoB 2.0 tool [16,18,23,41,42,43,44,45,46,47].

**Figure 3:**
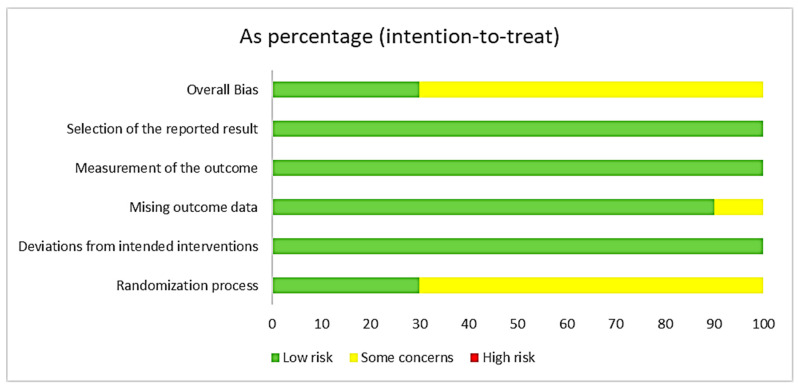
Risk of bias as a percentage (RoB 2).

The comprehensive assessment of the risk of bias for the included studies was meticulously conducted using Cochrane’s RoB 2.0 tool, which evaluates bias across multiple domains. The intention-to-treat (ITT) analysis approach was employed in evaluating the studies, aligning with the research plan to investigate the impacts of WBVE on CLBP patients. The assessment targeted the effect of assignment to the intervention at baseline, crucial for gauging the true effectiveness of WBVE. A summary of our findings is presented in Figure 2 and Figure 3.

Randomization Process: The randomization process is crucial in reducing selection bias in clinical trials. In this review, Jung et al. (2020) and Micke et al. (2021) demonstrated a low risk of bias in their randomization processes by using drawing lots and random number tables, respectively [41,44]. This methodological rigor suggests a high level of trust in the allocation of participants to intervention or control groups. Three studies also ensured low risk by maintaining concealed allocation sequences until participants were enrolled, adhering to best practices in clinical trials [23,41,44]. However, several studies presented some concerns regarding the randomization process. Specifically, seven of the included studies [16,18,42,43,45,46,47] did not sufficiently detail their random allocation methods, which may introduce bias and compromise the internal validity of the findings. The lack of description about the concealment of allocation particularly puts these studies at risk of bias. For instance, del Pozo-Cruz et al. (2011) used a random number table, and Wegener et al. (2019) utilized drawing lots for randomization, which are generally considered robust methods [16,46]. However, without proper details on allocation concealment, their risk of bias cannot be definitively deemed to be low. Kaeding et al. (2017) stratified randomization by sex and baseline pain intensity, a method that can help control for confounding variables but also requires careful concealment to prevent bias [42]. The computer-based randomization program used by Cigdem Karacay et al. (2022) represents a contemporary approach to random allocation, yet the lack of information on allocation concealment leaves some concern for potential bias [18].

Deviations from Intended Interventions: This area assesses the consistency and adherence to the intervention protocols. Remarkably, all included studies showed a low risk of bias in this domain, suggesting that the interventions were delivered as planned [16,18,23,41,42,43,44,45,46,47].

Missing Outcome Data: The integrity of the studies’ results heavily relies on complete outcome data. In our review, only one study was flagged with some concerns due to incomplete outcome data with high attrition rate [46], whereas the others were evaluated as low risk, indicating a comprehensive dataset was available for analysis [16,18,23,41,42,43,44,45,47].

Measurement of the Outcomes: Ensuring that outcomes are measured in a consistent, reliable manner is crucial for valid conclusions. All studies in our review were deemed to be low risk for this domain, which bolsters the credibility of the reported findings [16,18,23,41,42,43,44,45,46,47].

Selection of the Reported Result: The transparency with which results are reported affects the trustworthiness of the research. Our review found all studies to have a low risk of bias in reporting, underscoring a high level of transparency and accountability [16,18,23,41,42,43,44,45,46,47].

Overall Bias: When considering the overall risk of bias, the majority of studies were marked with some concerns primarily due to issues with randomization, allocation concealment and missing outcome data. Nevertheless, none of the studies were classified as having a high risk of bias, which speaks to the general quality and reliability of the included research.

### 3.6. PEDro Quality Assessment of Study Methodology

The methodological quality of the studies included in this systematic review was evaluated using the Physiotherapy Evidence Database (PEDro) scale. This scale is designed to assess the reliability and validity of clinical trials based on criteria that address various biases and methodological issues. A total of 12 studies were initially considered for inclusion, with their methodological quality assessed using the PEDro criteria, which includes aspects such as randomization, allocation concealment, and blinding of assessors (Table 5).

Out of the 12 studies, two were excluded due to low PEDro scores, indicating a potential risk of bias that could affect the validity of their results. Specifically, the studies by Sajadi et al. (2019) and Rittweger et al. (2002) were removed for scoring 3 and 4 out of 10, respectively, on the PEDro scale [48,49]. The remaining studies demonstrated a range of scores from 5 to 9. The study with the highest methodological quality was conducted by Micke et al. (2021), scoring 9 out of 10. This study stood out for its adherence to rigorous trial design principles, including proper randomization, allocation concealment, and blinding of outcome assessors, suggesting that the results are highly reliable [44]. On the other hand, studies by Maddalozzo et al. (2016), Yang et al. (2015), Rüger et al. (2023), and Wegner et al. (2019) scored the lowest among the included studies, each with a score of 5 [43,45,46,47].

The average PEDro score across the included studies was 6.5, reflecting a generally good level of methodological rigor. This average suggests that the systematic review’s findings are based on evidence from studies with a reasonable degree of reliability and validity. The PEDro scores inform the reader about the strength of the evidence presented, with higher scores correlating with stronger, more trustworthy evidence. This quality assessment is crucial for interpreting the review’s outcomes and for guiding future research and clinical application of WBVE in treating NSCLBP.

**Table 5:** Physiotherapy evidence database (PEDro) scale scores for each study included in the review.

Variables	Eligibility Criteria	Randomized Allocation	Concealed Allocation	Comparable at Baseline	Blinding of the Subjects	Blinding of the Therapist	Blinding of Assessors	Adequate Follow Up	Intention to Treat Analysis	Comparison between Groups	Point Estimates and Variability	Total Score
Ruger et al., 2023 [45]	1	1	0	1	0	0	0	1	0	1	1	5
Cigdem Karacay et al., 2022 [18]	1	1	0	1	0	0	1	1	1	1	1	7
Micke et al., 2021 [44]	1	1	1	1	0	1	1	1	1	1	1	9
Jung et al., 2020 [41]	1	1	1	1	0	1	0	1	0	1	1	7
Wang et al., 2019 [23]	1	1	1	1	0	0	1	1	1	1	1	8
Wegner et al., 2019 [46]	1	1	0	1	0	0	0	1	0	1	1	5
Sajadi et al., 2019 * [49]	1	1	0	0	0	0	0	0	0	1	1	3
Kaeding et al., 2017 [42]	1	1	0	1	0	0	1	1	1	1	1	7
Maddlozzo et al., 2016 [43]	1	0	0	1	0	0	0	1	1	1	1	5
Yang et al., 2015 [47]	1	1	0	1	0	0	0	1	0	1	1	5
del Pozo-Cruz et al., 2011 [16]	1	1	0	1	0	0	1	1	1	1	1	7
Rittweger et al., 2002 * [48]	1	1	0	1	0	0	0	0	0	1	1	4

* These studies were excluded from the review.

### 3.7. Findings of Certainty of Evidence

The detailed analysis of the certainty of evidence using the GRADE approach for the outcomes measured in the studies on WBVE NSCLBP reveals high certainty for most outcomes. For the ODI, VAS and NPRS for pain, RMDQ, proprioception (measured as the repositioning error), PILE, and the SF-36 QoL measure, no serious concerns were noted across the domains of risk of bias, inconsistency, indirectness, imprecision, and publication bias, indicating high-quality evidence. Balance outcomes, however, displayed a moderate level of certainty due to serious inconsistency potentially stemming from the different equipment used to measure outcomes. Overall, the evidence supports the effectiveness of WBVE for CLBP, as shown in the provided table (Table 6).

### 3.8. Effects of Intervention

Four studies reported improvement in VAS after the use of WBV [16,23,47]. Out of seven studies, six studies showed improvement in ODI score [16,23,43,45,46,47]. Three studies reported RMDQ for disability score and all the studies showed significant improvement after the intervention [16,18,42]. Out of five studies, two studies did not show significant improvement in balance score at one time point measurement [45]. The two studies showed significant improvement in proprioception after the intervention [23,41]. We included two studies for PILE; one study did not show a significant improvement in results [16], while another study showed improvement only in WBV compared with no intervention [18]. For SF-36, two studies showed significant improvement in both physical and mental components [46], while another study did not show any such changes [42] (Table 2 and Table 7).

### 3.9. Quantitative Analysis (Data Synthesis)

#### 3.9.1. Pain

Three studies with 90 participants in the WBV group and 88 in the control/alternative group were included for the pooled analysis, showing significant improvement in VAS value [SMD = −0.81, 95% CI (−1.11, −0.50), I^2^ = 0%, *p* < 0.01]. However, an insignificant large effect was revealed in the pooled analysis of NPRS, with a total of 95 participants in the experimental group and 80 in the control group, with a SMD of −1.14 [95% CI −2.40, 0.12, *p* = 0.08]. Although the direction of the mean difference suggests a potential benefit of WBV, the confidence interval crossing zero and the test for overall effect (Z = 1.78, *p* = 0.08) indicate that this result is not statistically significant.

#### 3.9.2. Disability

Seven studies with total of 253 participants in WBV group and 235 in the control/alternative group were included for the pooled analysis. One study was analyzed twice as it comprised three groups, so we separately compared WBV with both the groups (Ruger et al., 2023). A large significant reduction was seen in ODI score values [MD = −3.78, 95% CI (−5.27, −2.29), I^2^ = 24%, *p* < 0.01]. Three studies with total of 167 participants in the WBV group and 162 in the control/alternative group were included for the pooled analysis, showing a large significant reduction in RMDQ score values [MD = −1.43, 95% CI (−2.04, −0.82), I^2^ = 51%, *p* < 0.01].

### 3.10. Balance and Proprioception

Four studies with a total of 142 participants in the WBV group and 140 in the control/alternative group were included for the pooled analysis, showing moderate significant improvement in postural stability values [SMD = −0.28, 95% CI (−0.52, −0.05), I^2^ = 0%, *p* = 0.02].

Two studies with total of 115 participants in the WBV group and 113 in the control/alternative group were included for the pooled analysis, showing a significant large improvement in repositioning error [SMD = −4.20, 95% CI (−7.50, −0.89), I^2^ = 99%, *p* = 0.01].

### 3.11. Functional Performance and Quality of Life

Two studies with a total of 225 participants in the WBV group and 224 in the control/alternative group were included for the pooled analysis; they did not show improvement in PILE values [SMD = 0.25, 95% CI (−0.07, −0.58), I^2^ = 66%, *p* = 0.13].

**Figure 4:**
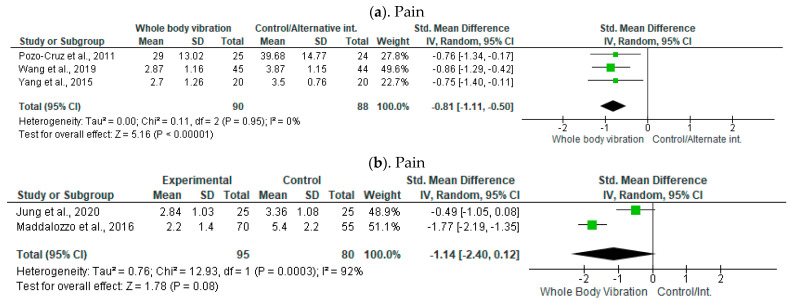

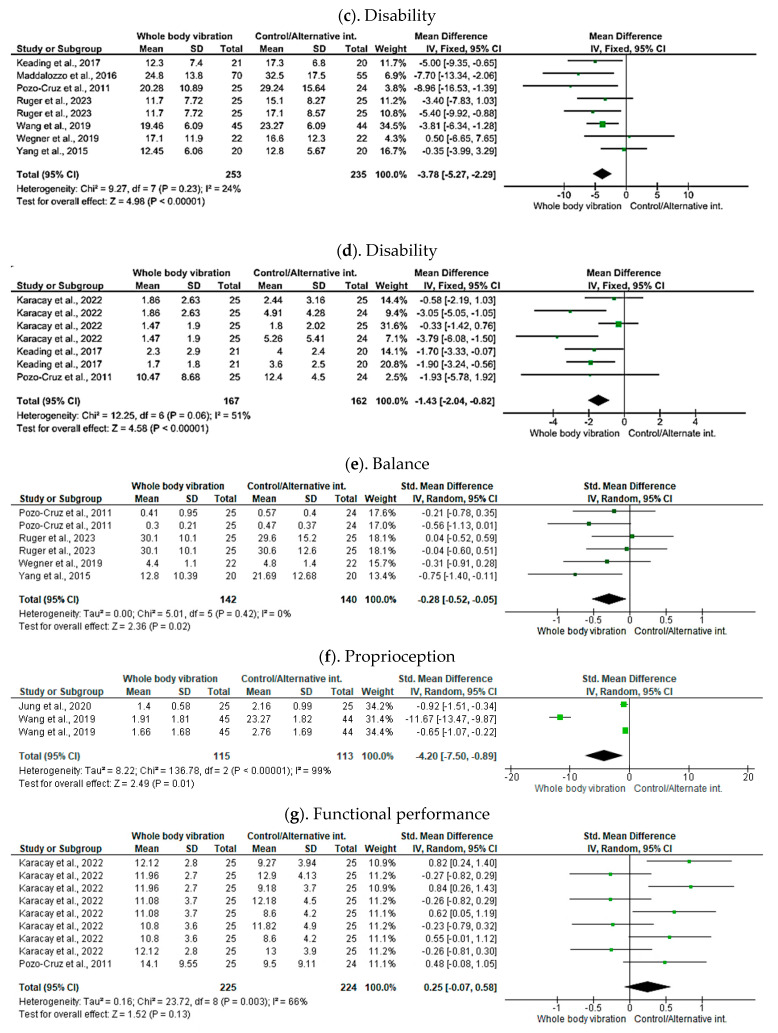

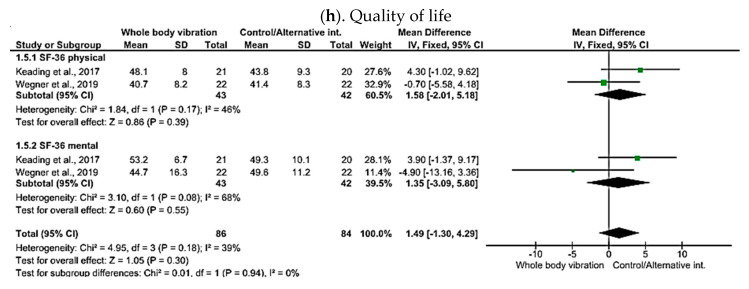
Forest plots of the effects of whole-body vibration exercise on non-specific chronic low back pain [16,18,23,41,42,43,45,46,47]. (**a**) Pain measured by Visual analog score; (**b**) Pain measured by Numerical pain rating scale; (**c**) Disability measured by Oswestry disability index; (**d**) Disability measured by Roland-Morris disability questionnaire; (**e**) Balance measured by postural stability test; (**f**) Proprioception measured by Lumbar repositioning; (**g**) Functional performance measured by Progressive isoinertial lifting evaluation test and (**h**) Quality of life measured by 36-Item short-form survey (SF-36). Note: The green squares indicate individual study effect sizes, horizontal lines represent confidence intervals, and the black diamond shows the pooled effect size from all studies.

Two studies with a total of 86 participants in the WBV group and 84 in the control/alternative group were included for the pooled analysis; they did not show improvement in SF-36 values [MD = 1.49, 95% CI (−1.30, −4.29), I^2^ = 39%, *p* = 0.30] (Figure 4).

## 4. Discussion

This systematic review and meta-analysis evaluated the efficacy of WBVE in managing NSCLBP. By examining 10 rigorously selected studies that compared WBVE interventions with non-vibration or control conditions in CLBP patients, our analysis found consistent evidence that WBVE can significantly reduce pain and disability, as measured by the VAS, ODI, and the RMDQ. Additionally, improvements were noted in proprioception and functional performance, including lifting capacity and balance. Our findings underscore the potential of WBVE to enhance muscle strength, proprioception, and overall QoL in individuals suffering from NSCLBP, providing valuable insights for physiotherapists and clinicians in the development of comprehensive treatment strategies for CLBP.

### 4.1. Pain

The systematic review and meta-analysis focusing on the impact of WBVE on individuals with NSCLBP reveals a significant improvement in pain scores, as evidenced by reductions in the VAS. This improvement aligns with previous literature that has documented the analgesic effects of WBVE, suggesting a multifaceted mechanism underlying these beneficial outcomes [26,27]. One of the primary theoretical frameworks that may explain the pain-relieving effects of WBVE is the gate control theory proposed by Melzack and Wall (1965). According to this theory, the vibration stimulus provided by WBVE activates large myelinated fibers, which can inhibit the transmission of pain signals carried by smaller pain fibers within the dorsal horn of the spinal cord [50]. This presynaptic inhibition acts as a ‘gate’, reducing the perception of pain. Furthermore, the simultaneous activation, by vibration, of sensory pathways associated with both muscle and skin is thought to produce more potent and sustained analgesic effects [51].

Additionally, the application of WBVE may promote muscle relaxation and alleviate pain associated with muscle tension. Previous studies have highlighted how increased muscle activity and blood flow, followed by a natural relaxation response after contraction, can lead to overall muscle relaxation [52,53]. This relaxation effect may further contribute to the reduction in pain scores observed following WBVE. Another important aspect of WBVE’s effectiveness in pain reduction is its potential to improve posture through the activation of trunk muscles [23]. This postural enhancement can reduce mechanical stress and tension on the passive structures of the trunk, as suggested by Jung et al. (2020), thereby contributing to pain alleviation [41]. Additionally, the non-noxious stimulus generated by vibration may decrease the activation of neurons in the spinothalamic tract and affect the synchronicity of neural signals reaching the cerebral hemisphere, leading to an increased pain threshold and reduced pain perception [54,55]. Integrating these findings, it becomes clear that the reduction in pain following WBVE interventions can be attributed to a complex interplay of physiological mechanisms. These include neural inhibition through the gate control theory, enhanced muscle relaxation and blood flow, improved postural control, and altered neural processing, which collectively contribute to the analgesic effects observed in NSCLBP patients.

### 4.2. Disability

The relationship between WBVE and its impact on disability in individuals with NSCLBP has acquired considerable interest within the rehabilitation sciences [18]. Disability, often quantified through measures such as the ODI, reflects the functional limitations and restrictions in daily living activities attributed to lower back pain [56]. A systematic review and meta-analysis of the literature indicate that WBVE can significantly improve disability outcomes in NSCLBP patients. This enhancement is likely attributable to the distinctive capability of WBVE to activate both musculoskeletal and nervous systems, leading to improvements in muscular strength, flexibility, and proprioception [57,58]. These elements are pivotal in diminishing disability associated with NSCLBP.

Mechanisms underlying the reduction of disability through WBVE involve several physiological pathways. Primarily, WBVE is thought to improve muscle function by enhancing muscle fiber activation and recruitment patterns, which, in turn, supports the stabilization of the lumbar spine and reduces mechanical stress on the back [59]. Enhanced muscle strength and coordination contribute to better functional performance and may reduce the disability associated with NSCLBP. Furthermore, WBVE has been shown to improve proprioceptive feedback, which is crucial for maintaining balance and stability during movement [60]. Enhanced proprioception can lead to improved motor control and reduced risk of falls and injuries, contributing to decreased disability scores [61,62].

### 4.3. Quality of Life

The impact of NSCLBP on QoL is profound, affecting physical, psychological, and social domains. Health-related QoL (HRQoL) assessments, such as the SF-36, provide a comprehensive overview of the patient’s perceived well-being and functioning [63]. While WBVE has shown promise in reducing pain and disability in NSCLBP patients, its effects on QoL are complex and multifaceted.

The relationship between pain, disability, and QoL is interconnected. As WBVE reduces pain and disability, one might expect corresponding improvements in QoL due to increased physical capabilities and reduced limitations in daily activities. However, the impact of WBVE on QoL may not always be directly positive, as evidenced by the mixed results in the literature. Some studies report significant improvements in both the physical and mental components of QoL following WBVE interventions [23,59], while others suggest minimal or no significant changes (Cardinale & Wakeling, 2005). These discrepancies may be attributed to various factors, including the study design, sample size, duration and frequency of WBVE, baseline QoL levels, and the specific QoL dimensions assessed.

Underlying mechanisms that could potentially enhance QoL through WBVE include the stimulation of the neuromuscular system, leading to improved physical function, increased endorphin release, and stress reduction [64]. Improved physical function can enhance an individual’s ability to perform daily activities, thus positively impacting the physical domain of QoL [65]. Endorphin release during physical activity is associated with mood elevation and pain reduction, potentially benefiting the mental and emotional aspects of QoL [66]. Furthermore, the engagement in WBVE may foster social interactions and support, contributing to improved social wellbeing [67]. Despite these potential benefits, the lack of significant effect observed in our pooled analysis may be explained by the limitations mentioned, such as small sample sizes and the heterogeneity in QoL reporting. Additionally, the perception of QoL is highly individual and can be influenced by numerous external factors beyond the scope of physical health improvements. It is also possible that the duration of the studies was not sufficient to observe significant changes in some QoL dimensions, which may require longer-term interventions to manifest.

### 4.4. Balance and Proprioception

Proprioception, a critical sensory feedback mechanism for maintaining balance and executing complex movements, plays a vital role in managing NSCLBP. The pooled analysis revealed significant improvement in proprioceptive and balance measures in our examination. A previous qualitative review also demonstrated that WBVE is effective in balance and proprioception in individuals with NSCLP [60].

The underlying mechanism attributed to these improvements involves the activation of proprioceptors, such as muscle spindles and Golgi tendon organs (GTOs), which enhance the sensory feedback loop required for maintaining postural equilibrium [68]. The application of WBV stimulates afferent pathways, leading to improved neuromuscular coordination [13,69]. This stimulation enhances the body’s ability to detect changes in joint position and movement, thereby refining motor control [70]. Enhanced proprioception aids in correcting postural deviations more efficiently, ultimately improving balance and stability [71]. This is particularly beneficial for individuals with NSCLBP, who often exhibit compromised postural control and proprioceptive accuracy due to pain and muscle weakness [41].

Moreover, WBV has been shown to facilitate the tonic vibration reflex (TVR), which triggers involuntary muscle contractions [72]. These contractions contribute to strengthening the muscles involved in postural support and balance [73], further stabilizing the lumbar region and reducing the likelihood of pain exacerbation [16,18,46]. Additionally, the simultaneous activation of both agonist and antagonist muscle groups during WBV can lead to a more balanced muscular system, reducing the predisposition for injury and enhancing overall stability [59,74].

### 4.5. Functional Capacity

The PILE assesses an individual’s capacity to perform repetitive lifting tasks, a critical aspect of functional performance, especially for those with CLBP [75]. Despite the proven benefits of WBV in enhancing muscle strength and endurance, the impact on lifting capacity as measured by PILE has shown mixed results. While some studies have indicated that WBV exercises can improve muscle performance, including that of the lumbar extensors and flexors, which are crucial for lifting tasks [76,77], our meta-analysis revealed an insignificant effect on lifting capacity.

This discrepancy could be attributed to the specificity of the vibration protocols used, the direction of the vibration, and the individual characteristics of the participants. Horizontal vibrations, for example, might more effectively trigger the TVR in the lumbar flexor and extensor muscles, potentially leading to improved lifting performance. However, the variation in study designs, vibration frequencies, and amplitudes makes it challenging to draw definitive conclusions. Furthermore, the ability of WBV to activate a larger portion of the motor neuron pool, including previously inactive motor units, suggests a theoretical basis for improvements in functional performance [27,78]. This enhanced motor unit recruitment could lead to more efficient force production during lifting tasks. Nevertheless, the current evidence underscores the need for further research to clarify the optimal vibration parameters and training protocols that would maximally benefit lifting capacity in individuals with NSCLBP.

### 4.6. Strength and Limitation

The strength of this systematic review with a meta-analysis relies on its adherence to rigorous methodological standards, including the use of the PEDro scale to ensure the inclusion of high-quality RCTs and the application of the updated version of the Cochrane Risk of Bias 2 tool alongside the GRADE approach to assess the certainty of evidence. This meticulous approach enhances the reliability of the findings and contributes to a more nuanced understanding of WBVE’s potential benefits across multiple outcomes, including pain, disability, balance, proprioception, functional performance, and QoL. Moreover, the study broadens the scope of investigation beyond the commonly reviewed outcomes by including functional performance and QoL among individuals with NSCLBP, areas that are critically important but often overlooked in similar research. This inclusion provides a more comprehensive overview of WBVE’s impact, offering valuable insights for clinicians and researchers interested in holistic treatment approaches.

However, this review is not without its limitations. The meta-analysis encompasses a relatively small number of studies with limited sample sizes, which may affect the generalizability of the results. The restriction to English-language RCTs further narrows the scope of the review, potentially omitting relevant studies published in other languages that could influence the overall findings. Additionally, the included studies exhibit considerable variability in WBVE treatment protocols, including differences in vibration frequency and amplitude. The lack of subgroup analyses exploring these variations limits our ability to draw specific conclusions about the optimal WBVE parameters for treating NSCLBP. Another critical limitation is the absence of an assessment for publication bias due to the small number of included studies, which could introduce a systematic overestimation or underestimation of the true effects of WBVE. Furthermore, our inclusion of studies with various combined interventions alongside WBVE, without a specific focus on the synergistic effects, may limit the specificity of our conclusions regarding the isolated impact of WBVE when used with one other specific treatment modality.

## 5. Future Perspective

Future research should aim to broaden the evidence base for the efficacy of WBVE in treating NSCLBP by including a more diverse range of randomized controlled trials, particularly those exploring the long-term effects of WBVE on isometric and isokinetic strength, muscle activation, and quality of life. Detailed analyses focusing on specific WBVE parameters, such as vibration frequency and amplitude, and their optimal application for different NSCLBP patient populations will be crucial. Additionally, studies with longer follow-up periods are needed to assess the sustainability of WBVE benefits over time and to understand its impact on the long-term management of NSCLBP. Addressing these areas will not only enhance our understanding of WBVE’s therapeutic potential but also contribute to the development of more targeted and effective rehabilitation strategies for individuals suffering from chronic low back pain.

## 6. Conclusions

This systematic review and meta-analysis critically evaluated the effectiveness of WBVE on individuals with NSCLBP, revealing that WBVE may offer significant benefits in terms of reducing pain, improving disability, enhancing balance and proprioception, and potentially affecting functional performance and QoL. While the findings suggest WBVE as a promising therapy for NSCLBP, they must be interpreted within the context of the study’s limitations, including the small number of included studies, sample size constraints, and variability in WBVE protocols.

## Figures and Tables

**Table 1:** Search strategy keywords.

Search Terms	Articles Found in Databases			
	PubMed	CENTRAL	Web of Science	Scopus
“Whole-body vibration”	2812	1523	4798	4779
“Whole-body vibration exercise”	532	892	1911	1906
“Whole-body vibration exercise” OR “Whole-body vibration”	709	1482	4797	4779
“Whole-body vibration exercise” AND “low back pain”	31	36	104	105
“Whole-body vibration” AND “chronic low back pain”	31	29	58	57
“Whole-body vibration “AND “chronic low back pain” AND “disability”	13	18	18	18
“Whole-body vibration” AND “chronic low back pain” AND “balance”	5	8	8	7
“Whole-body vibration” AND “chronic low back pain” AND “proprioception”	5	6	7	6
“Whole-body vibration” AND “chronic low back pain” AND “functional performance”	1	9	4	4
“Whole-body vibration” AND “chronic low back pain” AND “quality of life”	3	6	10	9
Total	4142	4009	11,715	11,670

Abbreviation: CENTRAL: Cochrane Central Register of Controlled Trials.

**Table 2:** Study Characteristics.

Study	Total Participants/Follow up	Diagnosis	Intervention and Comparator (Dosages)	Outcome Measures	Results
Ruger et al., 2023 [45]	*N* = 75/ Baseline and at 2nd week	NSCLBP above age of 18 year	Sensorimotor physiotherapy training Gallileo training Posturomed training 15 min/session, 6 total sessions	ODI, Posturography,	Statistically significant changes in ODI but no improvement in postural stability.
Cigdem Karacay et al., 2022 [18]	*N* = 74/ Baseline, 8 weeks and 20 weeks.	NSCLBP, 24 to 64 years of age	WBVE in 3 positions for 3 days/week for 8 weeks + classic lumbar home exercise Core stabilization + classic lumbar home exercise—3 days/week × 8 weeks William–Mckenzie home exercise—3 days/week × 8 weeks	VAS, RMDQ, PILE	Statistically significant difference in WBV group for VAS, RMDQ and PILE.
Micke et al., 2021 [44]	*N* = 240/ Baseline and 12 weeks	NSCLBP, ages 40–70 years	Electromyostimulation—1 day/week × 12 weeks WBVE—2 days/week for 12 weeks Warm up and circuit training for 1 day/week for 12 weeks	NPRS	Statistically significant and moderate improvement in NPRS all groups.
Jung et al., 2020 [41]	*N* = 50/ Baseline and after 12 weeks	NSCLBP aged between 10 and 19 years	Vibration—25 min/day, 3 day/week for 12 weeks Placebo: trunk stabilization exercise for same amount of time	Repositioning error, Lumbar kinematics Lumbar hip coordination NPRS	There was significant improvement in NPRS, Repositioning error.
Wang et al., 2019 [23]	*N* = 89/ Baseline and 12 weeks	NSCLBP from 3 months, pain score below 8	WBVE 3 times/week for 12 weeks Same exercise programme without vibration 3 times/week for 12 weeks	Visual analog scale Oswestry Disability Index Lumbar joint position sense SF-36	Statistically significant improvement in VAS, ODI, lumbar joint position sense, SF-36.
Wegener et al., 2019 [46]	*N* = 44/ Baseline and after the intervention	NSCLBP with age above 50 years	WBV Classic physiotherapy training All exercise were performed for 2 days/week for 6 weeks	Postural disability, ODI, SF-36	No significant improvement in ODI, SF-36.
Kaeding et al., 2017 [42]	*N* = 41/ Baseline and after 3 months	Chronic low back pain with minimum age of 18 year	WBVE 2.5 times/week for 3 months Control	RMDQ ODI, SF-36, static posturography	Significant improvement in RMDQ ODI and SF-36.
Maddalozzo et al., 2016 [43]	*N* = 125/ Baseline and end of the intervention	NSCLBP	Mckenzie exercise + WBV + traction Mckenzie exercise	ODI NPRS	Statistically significant improvement in both groups. However, more improvement in WBV+ traction group.
Yang et al., 2015 [47]	*N* = 40/ Baseline and 6 weeks.	CLBP with no neurological deficit from 12 weeks	WBV + 25 lumbar stability 3 days/week for 6 weeks 30 lumbar stability—3 days/week for 6 weeks	VAS KODI Static balance-fall index, posturography and postural sway	Significant improvement in WBV group for the fall index, VAS score, and ODI score. Spinal balance, VAS scale, and ODI score more improved in the control group than WBV.
del Pozo-Cruz et al., 2011 [16]	*N* = 50/ Baseline and 12 weeks.	Diagnosis of NSCLBP and minimum of 6 months of symptom	WBVE 2 times/week for 12 weeks Control	PILE RMDQ ODI VAS Biodex balance system	Statistically significant improvement in AP stability index, ODI, RMDQ, VAS, and PILE.

Abbreviation: NSCLBP: Non-specific chronic low back pain; CLBP: chronic low back pain; PILE: progressive iso-inertial lifting evaluation; RMDQ: Roland–Morris disability questionnaire; ODI: Oswestry disability index; KODI: Korean ODI, VAS: visual analog scale, WBV: whole-body vibration; AP: antero-posterior; SF-36: 36-Item short survey; and *N* = no. of participants.

**Table 3:** Whole-body vibration exercise regime in the included studies.

Authors and Year	Position of Application	Frequency (Hz)	Amplitude	Duration	Rest Time	Repetitions	Total Duration	Frequency of Sessions
Ruger et al., 2023 [45]	X	5–30 Hz	4.5 mm	X	X	X	15 min	Six sessions within 2 weeks
Cigdem Karacay et al., (2022) [18]	Knee bent to 120 Bridge Push up	25 Hz	2 mm	30 sec in 0–4 weeks 60 s in 4–8 week	30 s	2	4.5 min WBVE + 10 min warm up & cool down—0–4 weeks 7.5 min WBVE + 10 min warm up & cool down—0–4 weeks	3 days/week for 8 weeks
Micke et al., 2021 [44]	Dynamic cable squats Squats with arm extension Calf raises Static squats with arm movement, Static cable squats with calf raises	5–6 Hz 7–8 Hz 10 Hz 8–10 Hz 8 Hz	9 mm	60 s of oscillation	30 s	5–8 reps, 2 sets	15 min	2 days/week for 12 weeks
Jung et al., 2020 [41]	Single bridge Bridge Knee flex Plank Squat Bridge Side bridge	15 Hz	2 mm	60 s 60 s 60 s 60 s 90 s 90 s 90 s	30 s break after each exercise	X	5 min warm-up, 15 min whole-body vibration exercise and 5 min cool down	3 days/week for 12 weeks
Wang et al., 2019 [23]	Squat Kneeling Bridge Bridge with leg lift Bridge Knee flex Back release	9 Hz	X	90 s 60 s 90 s 60 s 60 s 90 s	30 30 30 30 30 30	2 2 2 2 2 2	5 min warm-up, 15 min whole-body vibration exercise and 5 min cool down	3 days/week for 12 weeks
Wegener et al., 2019 [46]	5 trunk stability exercises	5–12 Hz 12–20 20	X	1 min 1.5 min 2 min	X	X		Twice a week for 6 weeks
Kaeding et al., 2017 [42]	Basic position	10–30 Hz	1.5–3.5 mm	X	60 s	5	X	2.5/week for 3 months
Maddalozzo et al., 2016 [43]	Wall squats squats, and lunges + WBV traction	WBV traction table—20–30 Hz WBV platform—40–50 Hz	0.6–1.2 mm	X	X	X	X	X
Yang et al., 2015 [47]	Standing with slight flexion of knee joint and lumbar lordosis	18 Hz	X	5 min	X	X	5 min WBVE + 25 min lumbar stability training	3 days/week for 6 weeks
del Pozo-Cruz et al., 2011 [16]	Standing with the knee set at 120°	20 Hz	X	60 s 120 s180 s 240 s 360 s	30 s 30 s 30 s 30 s 0 s	6 3 2 2 1	6 min 6 min 6 min 8 min 6 min	2 days/week for 12 weeks

Abbreviations: X: not explained in the original study; Hz: Hertz; WBV: whole-body vibration and WBVE: whole-body vibration exercise.

**Table 4:** Outcome measures of included studies.

	NPRS	VAS	ODI	RMDQ	Balance	Proprioception	QoL	PILE
Ruger et al., 2023 [45]			✓		✓			
Cigdem Karacay et al., 2022 [18]		✓		✓				✓
Micke et al., 2021 [44]	✓							
Jung et al., 2020 [41]	✓					✓		
Wang et al., 2019 [23]		✓	✓			✓	✓	
Wegner et al., 2019 [46]			✓		✓		✓	
Kaeding et al., 2017 [42]			✓	✓	✓		✓	
Maddlozzo et al., 2016 [43]			✓					
Yang et al., 2015 [47]		✓	✓		✓			
del Pozo-Cruz et al., 2011 [16]		✓	✓	✓	✓			✓

Abbreviations: NPRS: numerical pain rating scale; VAS: visual analog scale; ODI: Oswestry disability index; RMDQ: Roland–Morris disability questionnaire, QoL: quality of life; PILE: progressive iso-inertial lifting evaluation; ✓: outcome is present in the study.

**Table 6:** Certainty of evidence (GRADE): whole-body vibration compared to control/alternative for non-specific chronic low back pain.

Certainty Assessment							Summary of Findings				
Participants (Studies) Follow-Up	Risk of Bias	Inconsistency	Indirectness	Imprecision	Publication Bias	Overall Certainty of Evidence	Study Event Rates (%)		Relative Effect (95% CI)	Anticipated Absolute Effects	
							With Control/Alternnate	With Whole-Body Vibration		Risk with Control/Alternnate	Risk Difference with Whole-Body Vibration
**ODI**											
488 (7 RCTs)	not serious	not serious	not serious	not serious	none	⨁⨁⨁⨁ High	235	253	-		MD **3.78 SD lower** (5.27 lower to 2.29 lower)
**VAS**											
228 (4 RCTs)	not serious	not serious	not serious	not serious	none	⨁⨁⨁⨁ High	113	115	-	-	SMD **0.81 SD lower** (1.11 lower to 0.5 lower)
**RMDQ**											
329 (3 RCTs)	not serious	not serious	not serious	not serious	none	⨁⨁⨁⨁ High	162	167	-		MD **1.43 SD lower** (2.04 lower to 0.82 lower)
**Repositioning error**											
228 (2 RCTs)	not serious	not serious	not serious	not serious	none	⨁⨁⨁⨁ High	113	115	-	-	SMD **4.2 lower** (7.5 lower to 0.89 lower)
**PILE**											
449 (2 RCTs)	not serious	not serious	not serious	not serious	none	⨁⨁⨁⨁ High	224	225	-	-	SMD **0.25 higher** (0.07 lower to 0.58 higher)
**Balance**											
242 (3 RCTs)	not serious	serious	not serious	not serious	none	⨁⨁⨁◯ Moderate	120	122	-	-	SMD **0.21 lower** (0.46 lower to 0.04 higher)
**SF-36**											
170 (2 RCTs)	not serious	not serious	not serious	not serious	none	⨁⨁⨁⨁ High	84	86	-		MD **1.49 SD higher** (1.3 lower to 4.29 higher)
**NPRS**											
85 (2 RCTs)	not serious	not serious	not serious	not serious	none	⨁⨁⨁⨁ High	80	95	-		SMD **1.14 higher** (2.40 higher to 0.12 lower)

Abbreviations: CI: confidence interval; MD: mean difference; SMD: standardized mean difference; RCT: randomized controlled trial; NPRS: numerical pain rating scale; VAS: visual analog scale; ODI: Oswestry disability index; RMDQ: Roland–Morris disability questionnaire; SF-36: short-form 36 health survey questionnaire; and PILE: progressive iso-inertial lifting evaluation.

**Table 7:** Summary table of the results.

Outcome Measure	Systematic Review	Meta-Analysis
NPRS	3/3 studies showed improvement	Large insignificant improvement
VAS	4/4 studies showed improvement	Significant large improvement
ODI	6/7 studies showed improvement	Significant large improvement
RMDQ	3/3 studies showed improvement	Significant large improvement
Balance	3/5 studies showed improvement	Significant moderate improvement
Proprioception	2/2 studies showed improvement	Significant large improvement
Quality of life (SF-36)	2/3 studies showed improvement	Not improved
PILE	1/2 studies showed improvement	Not improved

Abbreviations: NPRS: numerical pain rating scale; VAS: visual analog scale; ODI: Oswestry disability index; RMDQ: Roland–Morris disability questionnaire, SF-36: short-form 36 health survey questionnaire and PILE: progressive iso-inertial lifting evaluation.

## References

[1] Wu A., March L., Zheng X., Huang J., Wang X., Zhao J., Blyth F.M., Smith E., Buchbinder R., Hoy D. (2020). Global low back pain prevalence and years lived with disability from 1990 to 2017: Estimates from the Global Burden of Disease Study 2017. Ann. Transl. Med..

[2] Trapp W., Weinberger M., Erk S., Fuchs B., Mueller M., Gallhofer B., Hajak G., Kübler A., Lautenbacher S. (2015). A brief intervention utilising visual feedback reduces pain and enhances tactile acuity in CLBP patients. J. Back Musculoskelet. Rehabil..

[3] Manchikanti L., Singh V., Falco F.J., Benyamin R.M., Hirsch J.A. (2014). Epidemiology of low back pain in adults. Neuromodulation.

[4] Bagheri R., Takamjani I.E., Dadgoo M., Sarrafzadeh J., Ahmadi A., Pourahmadi M.R., Jafarpisheh A.S. (2017). A protocol for clinical trial study of the effect of core stabilization exercises on spine kinematics during gait with and without load in patients with non-specific chronic low back pain. Chiropr. Man. Ther..

[5] Russo M., Deckers K., Eldabe S., Kiesel K., Gilligan C., Vieceli J., Crosby P. (2018). Muscle Control and Non-specific Chronic Low Back Pain. Neuromodulation.

[6] Shokouhyan S.M., Davoudi M., Hoviattalab M., Abedi M., Bervis S., Parnianpour M., Brumagne S., Khalaf K. (2022). Distinction of non-specific low back pain patients with proprioceptive disorders from healthy individuals by linear discriminant analysis. Front. Bioeng. Biotechnol..

[7] Manojlović M. (2021). The efficiency of proprioceptive training in preventing injuries to team athletes: A systematic review. EQOL J..

[8] Zarei M., Eshghi S., Hosseinzadeh M. (2021). The effect of a shoulder injury prevention programme on proprioception and dynamic stability of young volleyball players; a randomized controlled trial. BMC Sports Sci. Med. Rehabil..

[9] Meier M.L., Vrana A., Schweinhardt P. (2019). Low Back Pain: The Potential Contribution of Supraspinal Motor Control and Proprioception. Neuroscientist.

[10] van Dieën J.H., Reeves N.P., Kawchuk G., van Dillen L.R., Hodges P.W. (2019). Motor Control Changes in Low Back Pain: Divergence in Presentations and Mechanisms. J. Orthop. Sports Phys. Ther..

[11] Tong M.H., Mousavi S.J., Kiers H., Ferreira P., Refshauge K., van Dieën J. (2017). Is there a relationship between lumbar proprioception and low back pain? A systematic review with meta-analysis. Arch. Phys. Med. Rehabil..

[12] Ghamkhar L., Kahlaee A.H. (2019). Pain and Pain-Related Disability Associated With Proprioceptive Impairment in Chronic Low Back Pain Patients: A Systematic Review. J. Manip. Physiol. Ther..

[13] Zhang J., Wang R., Zheng Y., Xu J., Wu Y., Wang X. (2021). Effect of Whole-Body Vibration Training on Muscle Activation for Individuals with Knee Osteoarthritis. BioMed Res. Int..

[14] Rittweger J. (2010). Vibration as an exercise modality: How it may work, and what its potential might be. Eur. J. Appl. Physiol..

[15] Cardinale M., Bosco C. (2003). The use of vibration as an exercise intervention. Exerc. Sport Sci. Rev..

[16] del Pozo-Cruz B., Hernández Mocholí M.A., Adsuar J.C., Parraca J.A., Muro I., Gusi N. (2011). Effects of whole body vibration therapy on main outcome measures for chronic non-specific low back pain: A single-blind randomized controlled trial. J. Rehabil. Med..

[17] Afridi B., Khan H., Akkol E.K., Aschner M. (2021). Pain Perception and Management: Where do We Stand?. Curr. Mol. Pharmacol..

[18] Cigdem Karacay B., Sahbaz T., Gurtekin B., Yildiz S., Ozcan E. (2022). Effectiveness of whole-body vibration exercise and core stabilization exercise in chronic non-specific low back pain: A randomized-controlled study. Turk. J. Phys. Med. Rehabil..

[19] Yin Y., Wang J., Yu Z., Zhou L., Liu X., Cai H., Sun J. (2022). Does whole-body vibration training have a positive effect on balance and walking function in patients with stroke? A meta-analysis. Front. Hum. Neurosci..

[20] Sá-Caputo D., Paineiras-Domingos L.L., Francisca-Santos A., Dos Anjos E.M., Reis A.S., Neves M.F.T., Oigman W., Oliveira R., Brandão A., Machado C.B. (2019). Whole-body vibration improves the functional parameters of individuals with metabolic syndrome: An exploratory study. BMC Endocr. Disord..

[21] Bartley E.J., Palit S., Fillingim R.B., Robinson M.E. (2019). Multisystem Resiliency as a Predictor of Physical and Psychological Functioning in Older Adults With Chronic Low Back Pain. Front. Psychol..

[22] Zhang M., Zhu W., He X., Liu Y., Sun Q., Ding H. (2022). Correlation between functional disability and quality of life among rural elderly in Anhui province, China: A cross-sectional study. BMC Public Health.

[23] Wang X.Q., Gu W., Chen B.L., Wang X., Hu H.Y., Zheng Y.L., Zhang J., Zhang H.Y., Chen P.J. (2019). Effects of whole-body vibration exercise for non-specific chronic low back pain: An assessor-blind, randomized controlled trial. Clin. Rehabil..

[24] Chen B., Dong Y., Guo J., Zheng Y., Zhang J., Wang X. (2019). Effects of Whole-Body Vibration on Lumbar-Abdominal Muscles Activation in Healthy Young Adults: A Pilot Study. Med. Sci. Monit..

[25] Li Q., Liu P., Wang Z., Li X. (2023). Vibration therapy to improve pain and function in patients with chronic low back pain: A systematic review and meta-analysis. J. Orthop. Surg. Res..

[26] Wang W., Wang S., Lin W., Li X., Andersen L.L., Wang Y. (2020). Efficacy of whole body vibration therapy on pain and functional ability in people with non-specific low back pain: A systematic review. BMC Complement Med. Ther..

[27] Remer F., Keilani M., Kull P., Crevenna R. (2023). Effects of whole-body vibration therapy on pain, functionality, postural stability, and proprioception in patients with subacute and chronic non-specific low back pain: A systematic review. Wien Med. Wochenschr.

[28] Montazeri A., Mousavi S.J., Preedy V.R., Watson R.R. (2010). Quality of Life and Low Back Pain. Handbook of Disease Burdens and Quality of Life Measures.

[29] Page M.J., McKenzie J.E., Bossuyt P.M., Boutron I., Hoffmann T.C., Mulrow C.D., Shamseer L., Tetzlaff J.M., Akl E.A., Brennan S.E. (2021). The PRISMA 2020 statement: An updated guideline for reporting systematic reviews. BMJ.

[30] Tawfik G.M., Dila K.A.S., Mohamed M.Y.F., Tam D.N.H., Kien N.D., Ahmed A.M., Huy N.T. (2019). A step by step guide for conducting a systematic review and meta-analysis with simulation data. Trop. Med. Health.

[31] Paolucci T., Attanasi C., Cecchini W., Marazzi A., Capobianco S.V., Santilli V. (2019). Chronic low back pain and postural rehabilitation exercise: A literature review. J. Pain Res..

[32] Balagué F., Mannion A.F., Pellisé F., Cedraschi C. (2012). Non-specific low back pain. Lancet.

[33] Jeffries L.J., Milanese S.F., Grimmer-Somers K.A. (2007). Epidemiology of adolescent spinal pain: A systematic overview of the research literature. Spine.

[34] Moseley A.M., Rahman P., Wells G.A., Zadro J.R., Sherrington C., Toupin-April K., Brosseau L. (2019). Agreement between the Cochrane risk of bias tool and Physiotherapy Evidence Database (PEDro) scale: A meta-epidemiological study of randomized controlled trials of physical therapy interventions. PLoS ONE.

[35] Cashin A.G., McAuley J.H. (2020). Clinimetrics: Physiotherapy Evidence Database (PEDro) Scale. J. Physiother..

[36] Sterne J.A.C., Savović J., Page M.J., Elbers R.G., Blencowe N.S., Boutron I., Cates C.J., Cheng H.-Y., Corbett M.S., Eldridge S.M. (2019). RoB 2: A revised tool for assessing risk of bias in randomised trials. BMJ.

[37] Schünemann H., Brożek J., Guyatt G., Oxman A. (2013). GRADE Handbook for Grading Quality of Evidence and Strength of Recommendations. Updated October 2013. The GRADE Working Group. https://www.rama.mahidol.ac.th/ceb/sites/default/files/public/pdf/journal_club/2017/GRADE%20handbook.pdf.

[38] Hultcrantz M., Rind D., Akl E.A., Treweek S., Mustafa R.A., Iorio A., Alper B.S., Meerpohl J.J., Murad M.H., Ansari M.T. (2017). The GRADE Working Group clarifies the construct of certainty of evidence. J. Clin. Epidemiol..

[39] Deeks J.J., Higgins J.P., Altman D.G., Group C.S.M. (2019). Analysing data and undertaking meta-analyses. Cochrane Handbook for Systematic Reviews of Interventions.

[40] Cohen J. (2013). Statistical Power Analysis for the Behavioral Sciences.

[41] Jung K.S., Jung J.H., In T.S., Cho H.Y. (2020). The Effectiveness of Trunk Stabilization Exercise Combined with Vibration for Adolescent Patients with Nonspecific Low Back Pain. Int. J. Environ. Res. Public Health.

[42] Kaeding T.S., Karch A., Schwarz R., Flor T., Wittke T.C., Kück M., Böselt G., Tegtbur U., Stein L. (2017). Whole-body vibration training as a workplace-based sports activity for employees with chronic low-back pain. Scand. J. Med. Sci. Sports.

[43] Maddalozzo G.F., Kuo B., Maddalozzo W.A., Maddalozzo C.D., Galver J.W. (2016). Comparison of 2 Multimodal Interventions With and Without Whole Body Vibration Therapy Plus Traction on Pain and Disability in Patients With Nonspecific Chronic Low Back Pain. J. Chiropr. Med..

[44] Micke F., Weissenfels A., Wirtz N., von Stengel S., Dörmann U., Kohl M., Kleinöder H., Donath L., Kemmler W. (2021). Similar Pain Intensity Reductions and Trunk Strength Improvements Following Whole-Body Electromyostimulation vs. Whole-Body Vibration vs. Conventional Back-Strengthening Training in Chronic Non-specific Low Back Pain Patients: A Three-Armed Randomized Controlled Trial. Front. Physiol..

[45] Rüger A., Laudner K., Delank K.S., Schwesig R., Steinmetz A. (2023). Effects of Different Forms of Sensorimotor Training on Postural Control and Functional Status in Patients with Chronic Low Back Pain. J. Pers. Med..

[46] Wegener V., Rarack S., Tiffe T., Grill E., Melcher C., Birkenmaier C., Jansson V., Wegener B. (2019). Effects of Whole Body Vibration Therapy and Classic Physiotherapy on Postural Stability in People With Back Pain: A Randomized Trial. Clin. Spine Surg..

[47] Yang J., Seo D. (2015). The effects of whole body vibration on static balance, spinal curvature, pain, and disability of patients with low back pain. J. Phys. Ther. Sci..

[48] Rittweger J., Just K., Kautzsch K., Reeg P., Felsenberg D. (2002). Treatment of chronic lower back pain with lumbar extension and whole-body vibration exercise: A randomized controlled trial. Spine.

[49] Sajadi N., Bagheri R., Amiri A., Maroufi N., Shadmehr A., Pourahmadi M. (2019). Effects of Different Frequencies of Whole Body Vibration on Repositioning Error in Patients With Chronic Low Back Pain in Different Angles of Lumbar Flexion. J. Manip. Physiol. Ther..

[50] Melzack R., Wall P.D. (1965). Pain mechanisms: A new theory. Science.

[51] Comitato A., Bardoni R. (2021). Presynaptic Inhibition of Pain and Touch in the Spinal Cord: From Receptors to Circuits. Int. J. Mol. Sci..

[52] Elfering A., Burger C., Schade V., Radlinger L. (2016). Stochastic resonance whole body vibration increases perceived muscle relaxation but not cardiovascular activation: A randomized controlled trial. World J. Orthop..

[53] Elfering A., Zahno J., Taeymans J., Blasimann A., Radlinger L. (2013). Acute effects of stochastic resonance whole body vibration. World J. Orthop..

[54] Kim H., Kwon B.S., Park J.W., Lee H., Nam K., Park T., Cho Y., Kim T. (2018). Effect of Whole Body Horizontal Vibration Exercise in Chronic Low Back Pain Patients: Vertical Versus Horizontal Vibration Exercise. Ann. Rehabil. Med..

[55] Yam M.F., Loh Y.C., Tan C.S., Khadijah Adam S., Abdul Manan N., Basir R. (2018). General Pathways of Pain Sensation and the Major Neurotransmitters Involved in Pain Regulation. Int. J. Mol. Sci..

[56] Bang A.A., Bhojraj S.Y., Deshmukh M., Kalkotwar S., Joshi V.R., Yarmal T., Kalkonde Y., Bang A.T. (2021). Activity limitation and disability due to pain in back and extremities in rural population: A community-based study during a period of twelve months in rural Gadchiroli, India. J. Glob. Health.

[57] Bonanni R., Cariati I., Romagnoli C., D’Arcangelo G., Annino G., Tancredi V. (2022). Whole Body Vibration: A Valid Alternative Strategy to Exercise?. J. Funct. Morphol. Kinesiol..

[58] Rasti E., Rojhani-Shirazi Z., Ebrahimi N., Sobhan M.R. (2020). Effects of whole body vibration with exercise therapy versus exercise therapy alone on flexibility, vertical jump height, agility and pain in athletes with patellofemoral pain: A randomized clinical trial. BMC Musculoskelet. Disord..

[59] Rittweger J., Mutschelknauss M., Felsenberg D. (2003). Acute changes in neuromuscular excitability after exhaustive whole body vibration exercise as compared to exhaustion by squatting exercise. Clin. Physiol. Funct. Imaging.

[60] Tariq N., Khan Z., Veqar Z. (2023). Effect of Whole-Body Vibration on Balance or Proprioception in Nonspecific Chronic Low Back Pain: A Systematic Review. J. Chiropr. Med..

[61] Winter L., Huang Q., Sertic J.V.L., Konczak J. (2022). The Effectiveness of Proprioceptive Training for Improving Motor Performance and Motor Dysfunction: A Systematic Review. Front. Rehabil. Sci..

[62] Yu Y., Chen Y., Lou T., Shen X. (2021). Correlation Between Proprioceptive Impairment and Motor Deficits After Stroke: A Meta-Analysis Review. Front. Neurol..

[63] Lins L., Carvalho F.M. (2016). SF-36 total score as a single measure of health-related quality of life: Scoping review. SAGE Open Med..

[64] Xie Y., Wu Z., Sun L., Zhou L., Wang G., Xiao L., Wang H. (2021). The Effects and Mechanisms of Exercise on the Treatment of Depression. Front. Psychiatry.

[65] Marquez D.X., Aguiñaga S., Vásquez P.M., Conroy D.E., Erickson K.I., Hillman C., Stillman C.M., Ballard R.M., Sheppard B.B., Petruzzello S.J. (2020). A systematic review of physical activity and quality of life and well-being. Transl. Behav. Med..

[66] Mahindru A., Patil P., Agrawal V. (2023). Role of Physical Activity on Mental Health and Well-Being: A Review. Cureus.

[67] Davis A.J., MacCarron P., Cohen E. (2021). Social reward and support effects on exercise experiences and performance: Evidence from parkrun. PLoS ONE.

[68] Blecher R., Heinemann-Yerushalmi L., Assaraf E., Konstantin N., Chapman J.R., Cope T.C., Bewick G.S., Banks R.W., Zelzer E. (2018). New functions for the proprioceptive system in skeletal biology. Philos. Trans. R Soc. Lond. B Biol. Sci..

[69] Li K.-y., Cho Y.-j., Chen R.-s. (2021). The Effect of Whole-Body Vibration on Proprioception and Motor Function for Individuals with Moderate Parkinson Disease: A Single-Blind Randomized Controlled Trial. Occup. Ther. Int..

[70] Han J., Waddington G., Adams R., Anson J., Liu Y. (2016). Assessing proprioception: A critical review of methods. J. Sport Health Sci..

[71] Aman J.E., Elangovan N., Yeh I.L., Konczak J. (2014). The effectiveness of proprioceptive training for improving motor function: A systematic review. Front. Hum. Neurosci..

[72] Kalaoğlu E., Faruk Bucak Ö., Kökçe M., Özkan M., Çetin M., Atasoy M., Aytüre L., Karacan İ. (2023). High-frequency whole-body vibration activates tonic vibration reflex. Turk. J. Phys. Med. Rehabil..

[73] Ivanenko Y., Gurfinkel V.S. (2018). Human Postural Control. Front. Neurosci..

[74] Aminian-Far A., Hadian M.R., Olyaei G., Talebian S., Bakhtiary A.H. (2011). Whole-body vibration and the prevention and treatment of delayed-onset muscle soreness. J. Athl. Train..

[75] Mayer T.G., Barnes D., Nichols G., Kishino N.D., Coval K., Piel B., Hoshino D., Gatchel R.J. (1988). Progressive isoinertial lifting evaluation. II. A comparison with isokinetic lifting in a disabled chronic low-back pain industrial population. Spine.

[76] Torvinen S., Kannus P., Sievänen H., Järvinen T.A., Pasanen M., Kontulainen S., Järvinen T.L., Järvinen M., Oja P., Vuori I. (2002). Effect of four-month vertical whole body vibration on performance and balance. Med. Sci. Sports Exerc..

[77] Xiong W., Liu X. (2023). Effects of whole-body vibration training combined with KAATSU training on lower limb joint muscle strength in older women. Front. Physiol..

[78] Li Y., Yan L., Hou L., Zhang X., Zhao H., Yan C., Li X., Li Y., Chen X., Ding X. (2023). Exercise intervention for patients with chronic low back pain: A systematic review and network meta-analysis. Front. Public Health.

